# Supplementary figures and images for: Selective demethylation of two CpG sites causes postnatal activation of the Dao gene and consequent removal of d-serine within the mouse cerebellum
Source: Clin Epigenetics. 2019 Oct 28;11:149. doi: 10.1186/s13148-019-0732-z (PMC6819446; doi:10.1186/s13148-019-0732-z)

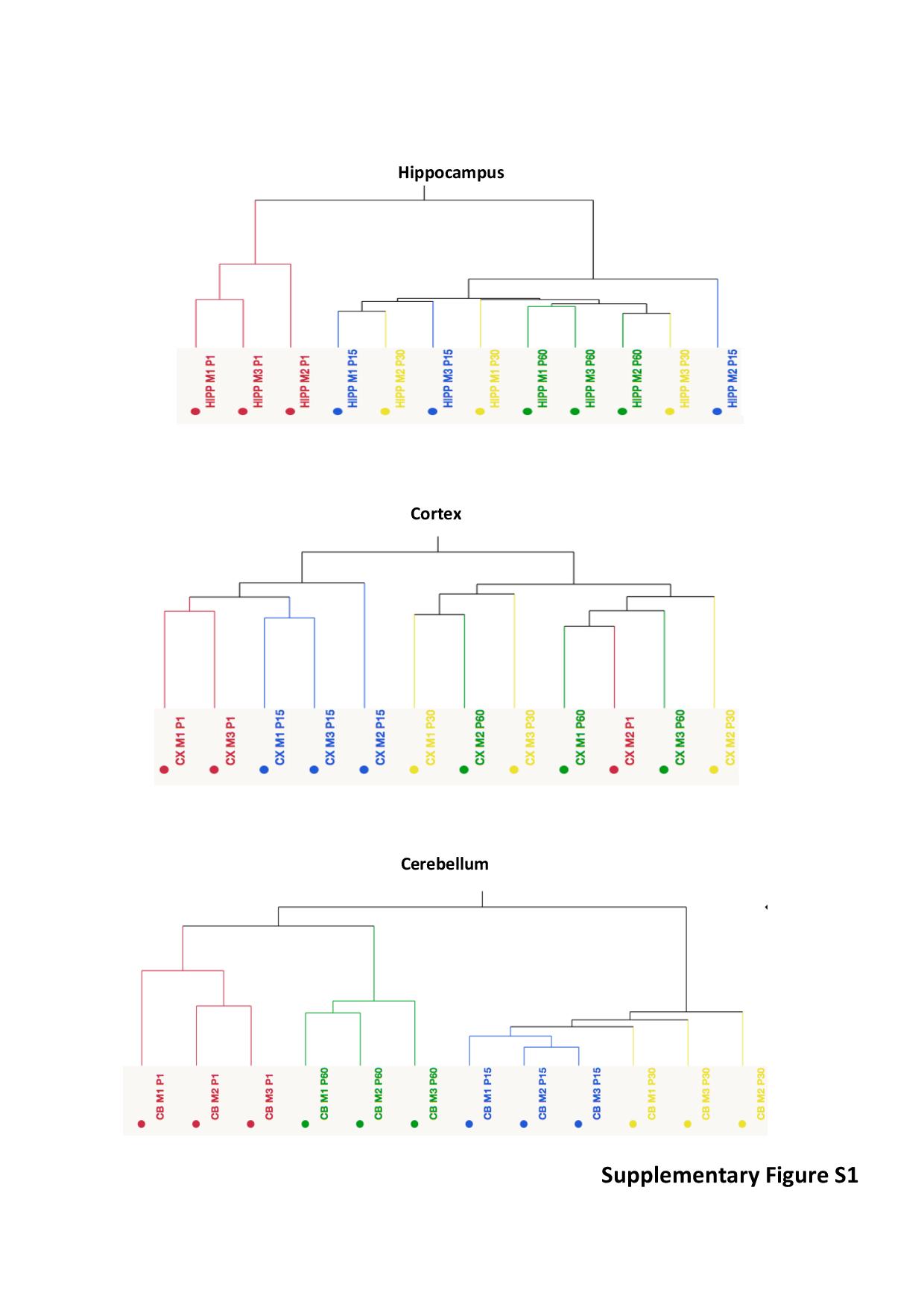

Supplement: Supplementary file 1 — Figure S1. Ddo epiallelic distribution analysis in all analyzed brain regions during ontogenesis. Hierarchical cluster based on the epiallelic composition of each developmental stage is presented for HIPP, CX and CB. Developmental stages are indicated with different colors (P1 = red; P15 = blue; P30 = yellow; P60 = green). (JPG 87 kb) [file 13148_2019_732_MOESM1_ESM.jpg]

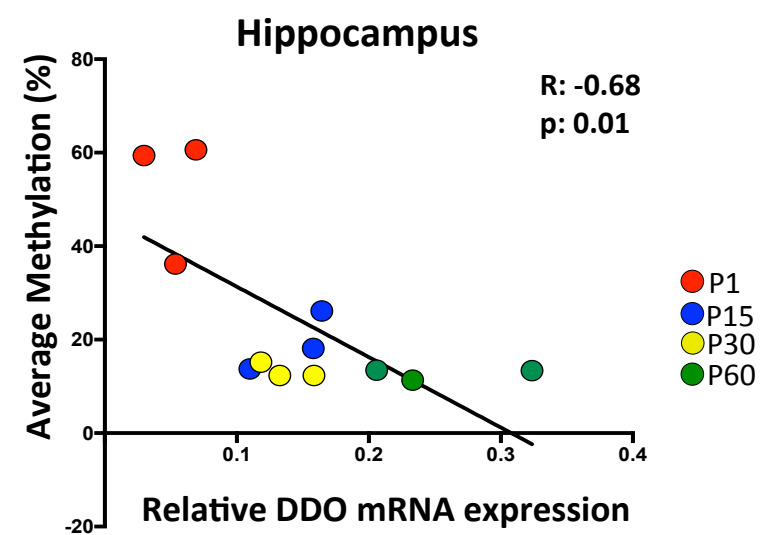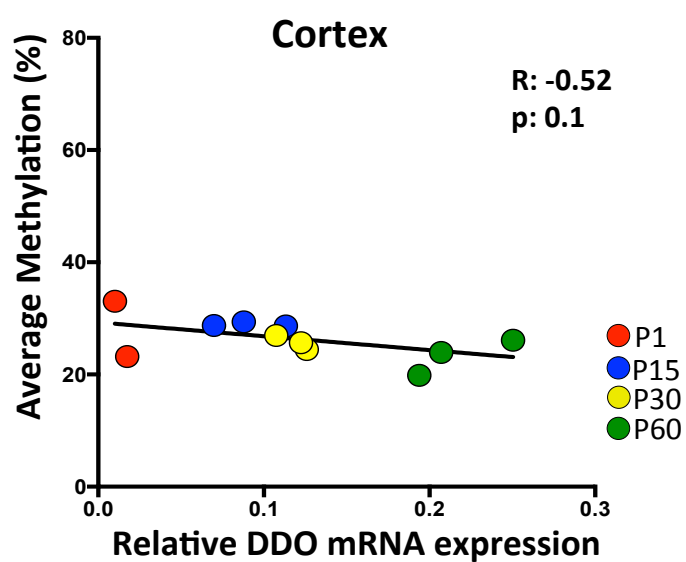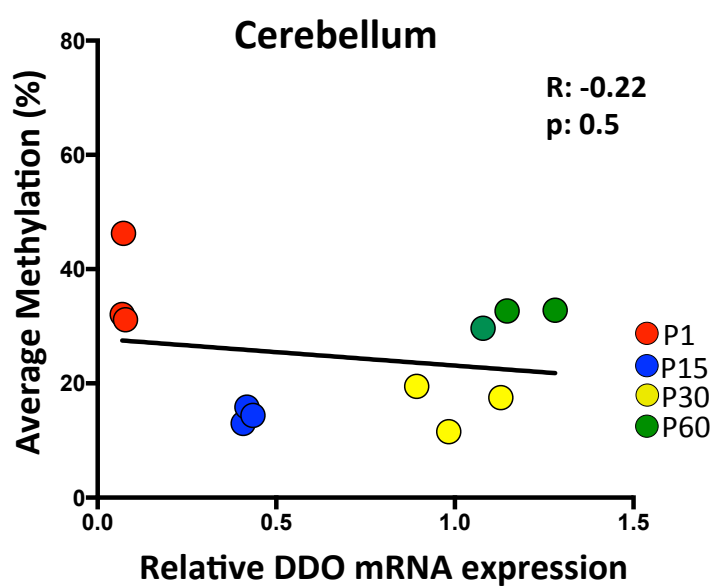

Supplementary Figure S2

Supplement: Supplementary file 2 — Figure S2. Regression analysis associating the degree of methylation with mRNA expression levels of the Ddo gene. Regression analysis associating DNA methylation and mRNA expression of Ddo gene is shown in each analyzed brain area during development. Ddo mRNA expression is normalized to the mean values for two housekeeping genes and expressed as 2–∆Ct values. The three mice for each time point (P1 = red; P15 = blue; P30 = yellow; P60 = green) are indicated. Statistical analyses were performed using Pearson correlation. * p ≤ 0.05 (PDF 86 kb) [file 13148_2019_732_MOESM2_ESM.pdf]
